# Data on body mass, glucose tolerance and bone phenotype of mice with osteogenesis imperfecta on long-term low-fat and high-fat diets

**DOI:** 10.1016/j.dib.2022.107961

**Published:** 2022-02-16

**Authors:** Josephine T. Tauer, Iris Boraschi-Diaz, Svetlana V. Komarova

**Affiliations:** a Faculty of Dental Medicine and Oral Health Sciences, McGill University, 2001 McGill College Avenue, Montreal, Quebec H3A 1G1, Canada; b Shriners Hospital for Children-Canada, 1003 Boulevard Decarie, Montreal, Quebec H4A 0A9, Canada; c Department of Pediatrics, McGill University, 1001 Boulevard Decarie, Montreal, Quebec H4A 3J1, Canada

**Keywords:** Animal model, Glucose intolerance, Osteogenesis imperfecta, Body mass, Bone phenotype, High-fat diet, FVB, OI, Osteogenesis imperfecta, CT, Computed tomography, WT, Wild-type, HFD, High-fat/low-sugar diet, LFD, Low-fat/low-sugar diet, GTT, Glucose tolerance test, BAT, Brown adipose tissue, WAT, White adipose tissue, AUC, Area under the curve, SEM, Standard error of mean, BMD, Bone mineral density, Tb.Sp., Trabecular separation, Trab.BMD, Trabecular bone mineral density, BV/TV, bone volume/tissue volume, PBS, phosphate-buffered saline

## Abstract

Male and female mice with a dominant severe bone fragility disorder, osteogenesis imperfecta, and their wild-type littermates (FVB background) were challenged with a long-term (26 weeks) high-fat diet to evaluate the development of obesity and glucose intolerance. Here we present data for the measurements of body mass, the outcome of glucose tolerance tests during the long-term diet, as well as organ weights and bone phenotype at the end of the study. Interpretation of the data and further in-depth analysis can be found in the article “Male but not female mice with severe osteogenesis imperfecta are partially protected from high-fat diet-induced obesity.” by Tauer JT, Boraschi-Diaz I, Al Rifai O, Rauch F, Ferron M, Komarova SV, published in Molecular Genetics and Metabolism. The data presented here demonstrate individual mouse outcomes of long-term diet experiments that can be reused for comparative studies of diet-induced changes in wild-type mice on different backgrounds and different mouse models of osteogenesis imperfecta.

## Specifications Table

**Table:** 

Subject	Endocrinology, Diabetes and Metabolism
Specific subject area	Long-term high-fat diet in a mouse model with dominant severe osteogenesis imperfecta.
Type of data	Graph, Figure
How data were acquired	Body mass: portable compact balance (OHAUS Corporation, Model CS200,Parsippany, US); Glucose tolerance tests: ONETOUCH VerioFlex glucometer (LifeScan Europe, Zug, Switzerland); Organ weights: analytical balance (Denver Instrument GmbH, Model P-114, Goettingen, Germany); long-bone length: digital caliper (Starrett, Model EC799A-6/150, Athol, US); *ex vivo* micro-computed tomography: Skyscan 1272 (Bruker, MA, US); Micro-computed tomography image analysis: Skyscan CT Analyser (Version 1.16.1.0, Bruker, MA, US); three-point bending: MaterialsTesting System Model 5943 (INSTRON, Norwood, MA, USA)
Data format	Raw and analyzed
Parameters for data collection	Male and female wild-type (WT) and *Col1a1*^Jrt/+^mice (OI) were randomly assigned to treatment with either a high-fat/low-sugar diet or control low-fat/low-sugar diet starting at an age of 4 weeks until 30 weeks of age (diet length: 26 weeks). Animals were housed under standard conditions with 12 h alternating light and dark cycle and unrestricted access to water and food.
Description of data collection	Body mass was recorded weekly. Glucose tolerance tests were performed every four weeks. At the end of the study, mice were terminated, and soft tissues were isolated, weighted and snap-frozen in liquid nitrogen. Right and left femora were isolated, length measured, and stored at −20 °C in phosphate buffered saline-soaked gauze until testing. Right femurs were collected for *ex vivo* micro-computed tomography and three-point bending test.
Data source location	Institution: McGill University, Shriners Hospital for Children-Canada City/Town/Region: Montreal, Quebec Country: Canada
Data accessibility	Link: https://data.mendeley.com/datasets/np7kpnk9t4/1 DOI:10.17632/np7kpnk9t4.1
Related research article	J.T. Tauer, I. Boraschi-Diaz, O. Al Rifai, F. Rauch, M. Ferron, S.V. Komarova. Male but not female mice with severe osteogenesis imperfecta are partially protected from high-fat diet-induced obesity. Mol Genet Metab. 2021 Jun;133(2):211–221. doi:10.1016/j.ymgme.2021.03.014. Epub 2021 Mar 30

## Value of the Data

• The presented data are useful for researchers interested in long-term high-fat diet-induced metabolic response in a mouse model of osteogenesis imperfecta and their FVB wild-type littermates.
• The presented data are beneficial for researchers investigating the influence of diet-induced obesity on bone phenotype in mice.
• The presented data are valuable to understand the relationship of diet-induced obesity on glucose metabolism and osseous phenotype in relation to sex in mice.
• The data presented might be reused for comparative studies of diet-induced changes in wild-type mice on different backgrounds and in different mouse models of osteogenesis imperfecta.

## 1 Data Description

Interpretation of the data and further in-depth analysis can be found here [1]. All raw individual mouse outcomes of long-term diet experiments can be found in the excel file available by the Mendeley database [2]. The excel file contains individual mouse data about body mass development (excel sheet 1), performed glucose tolerance tests (GTT) (excel sheet 2), calculated GTT-related area under the curve (excel sheet 3), fasted glucose levels (excel sheet 4), absolute organ weights (excel sheet 5), and femoral structural (excel sheet 6) and biomechanical parameters (excel sheet 7).

**Fig. 1:**
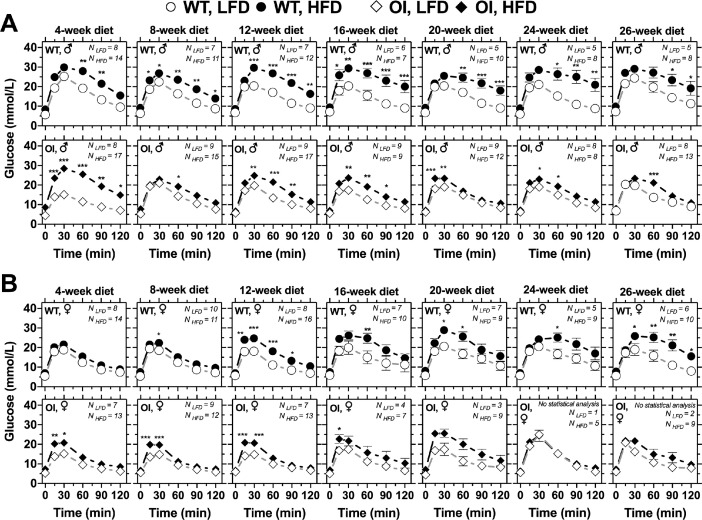
*Glucose tolerance curves after 4-, 8-, 12-, 16-, 20-, 24- and 26-weeks of low-fat (LFD, white symbols) or high-fat (HFD, black symbols) diet in male (A) and female (B) WT (circle) and OI mice (rhombus)*. Data represent mean ± standard error of mean (SEM). Statistical analysis: two-way ANOVA of repeated measures followed by Bonferroni post-test: * *p* < 0.05, ** *p* < 0.01, *** *p* < 0.001.

**Fig. 2:**
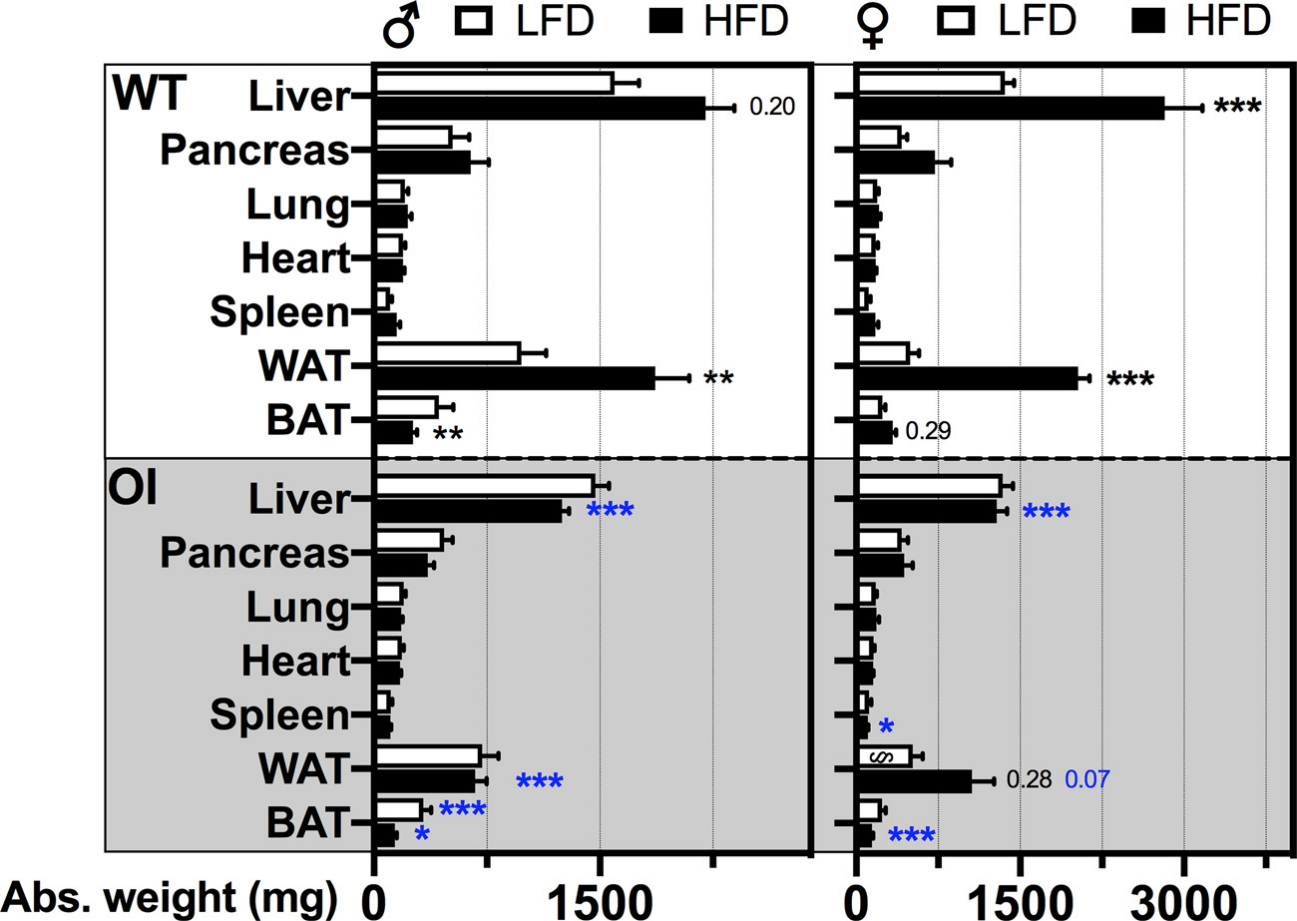
*Absolute organ weights in WT and OI mice after long-term low-fat (LFD) or high-fat (HFD) diet*. Data represent mean ± standard error of mean (SEM). Animal numbers were as follows: male WT _LFD_*n* = 6; male WT _HFD_*n* = 9; female WT _LFD_*n* = 7; female WT _HFD_*n* = 10; male OI _LFD_*n* = 7; male OI _HFD_*n* = 13; female OI _LFD_*n* = 2; female OI _HFD_*n* = 9. Statistical analysis: two-way ANOVA with Bonferroni post-test. Significances below 0.99 are indicated by either p-value or significance symbol as * *p* < 0.05, ** *p* < 0.01, *** *p* < 0.001. Further, significances are color coded and demonstrate diet effect: black, and genotype effect: blue. §, Statistical analysis might be compromised by low animal number of this group.

**Fig. 3:**
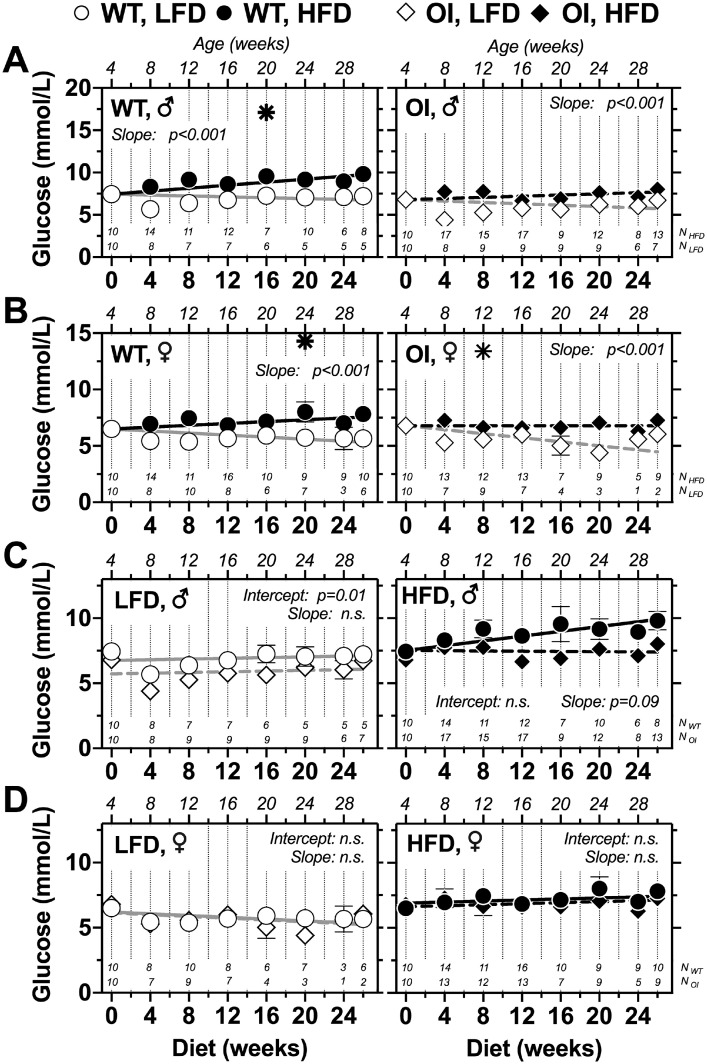
*Regression analysis of fasted glucose levels during long-term low-fat diet (LFD) or high-fat diet (HFD) in male and female WT (circle) and OI (rhombus) mice*. Fasted glucose levels refer to time point 0 min of performed glucose tolerance tests. Data represent mean ± standard error of mean (SEM). Statistical analysis: (A, B) non-linear regression analysis of LFD-fed and HFD-fed mice with the same genotype and evaluation of statistical differences between curve slopes. (C, D) Non-linear regression analysis of WT and OI mice on either LFD or and HFD with evaluation of statistical differences between intercept and slope of the curves. Star symbol: statistical outliers, detected by ROUT method [3], not included in regression analysis.

**Fig. 4:**
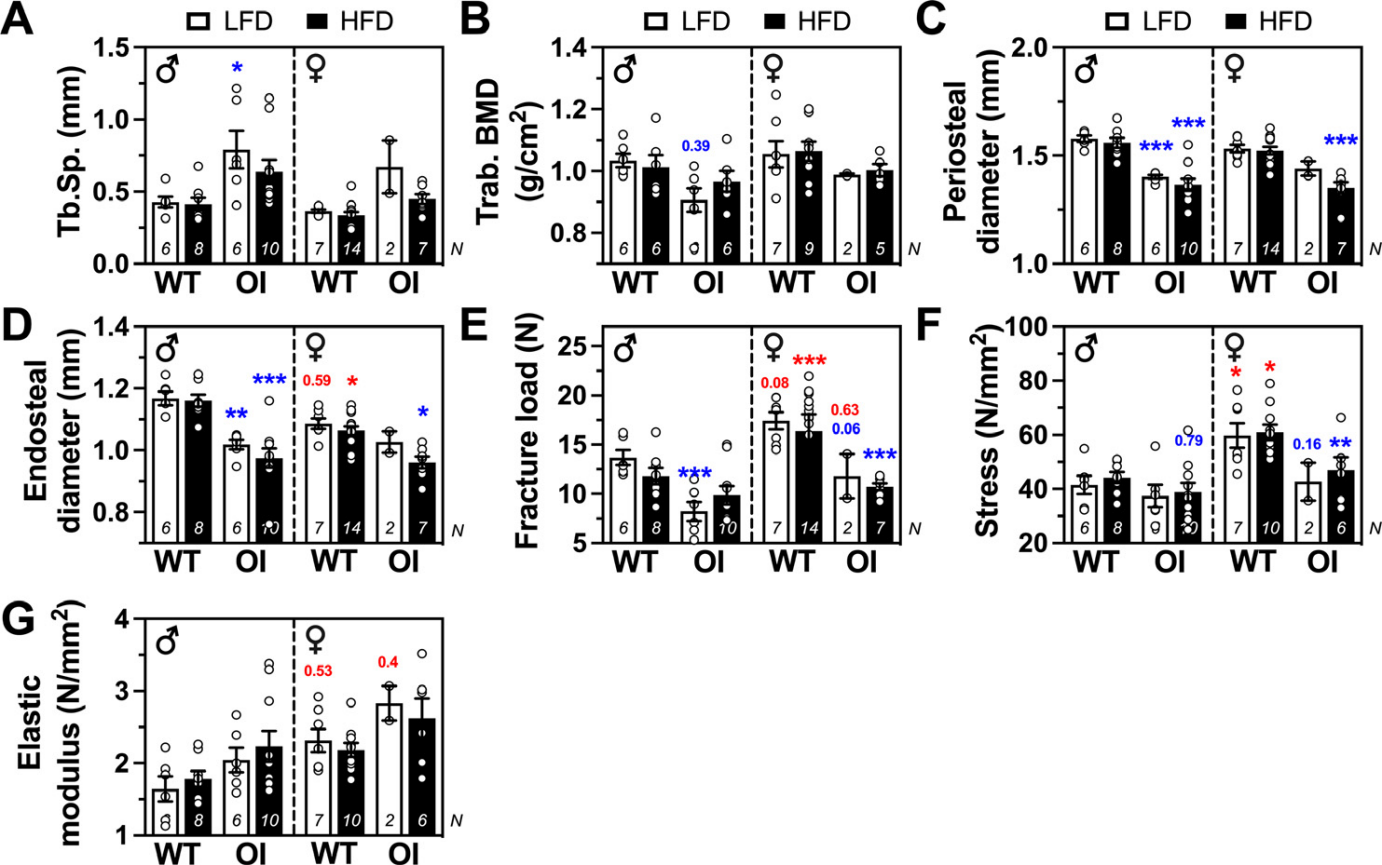
*Femoral structural (A-D) and biomechanical parameters (E–G) after long-term diets in male and female WT and OI mice*. Data represent mean ± standard error of mean (SEM). LFD, low-fat diet; HFD, high-fat diet; Tb.Sp., trabecular separation; trab. BMD, trabecular bone mineral density. Statistical analysis: three-way ANOVA with Bonferroni post-test. Significances below 0.99 are indicated by either p-value or significance symbol as* *p* < 0.05, ** *p* < 0.01, *** *p* < 0.001. Further, significances are color coded and demonstrate diet effect: black, genotype effect: blue, sex effect: red.

In brief, data describe long-term diet-induced weight gain in WT and OI mice and its possible effects on glucose homeostasis and bone properties. Fig. 1 depicts glucose homeostasis measurements during 26-week diet-intervention in male and female WT and OI mice. Fig. 2 depicts absolute organ weights of liver, pancreas, lung, heart, spleen, brown adipose tissue (BAT), and white adipose tissue (WAT) in male and female WT and OI mice at the end of the study. Fig. 3 depicts regression analysis of fasted glucose levels during long-term diet regimen in male and female WT and OI mice. Fig. 4 depicts selected femoral structural and mechanical properties in male and female WT and OI mice at the end of the study. Further, femoral structural and mechanical parameters can be found here [1] and in the Mendeley database.

## 2 Experimental Design, Materials and Methods

### 2.1 Animal experiment, treatment, and data collection

Experimental design and treatment are described here [1]. In brief, the *Col1a1^Jrt/+^*mouse on FVB background was used as mouse model for dominant moderate-to-severe Osteogenesis imperfecta (OI) [4,5]. *Col1a1^Jrt/+^*mice carry a splice site mutation in exon 9 of the *COL1A1* gene leading to an 18-amino acid deletion in the collagen type I α1 chain [4]. *Col1a1^Jrt/+^*mice are smaller in size and demonstrate OI bone phenotype with shorter long bones, lower bone volume/tissue volume (BV/TV), and development of spontaneous fractures [4]. The breeding colony was maintained at the Animal Care Facility of the Shriners Hospitals for Children-Canada. Animals were on a 12 h alternating light and dark cycle and had unrestricted access to water and food. Prior to long-term diet regimen, mice were fed a regular chow (Rodent chow #5075, Charles River Laboratories). Male and female wild-type (WT) and OI mice were challenged with either a high-fat/low-sugar diet (HFD; Teklad Custom Diet, #TD.06414) or control low-fat/low-sugar diet (LFD; Teklad Custom Diet, #TD.180127), starting at an age of 4 weeks until 30 weeks of age (diet length: 26 weeks). Diets were purchased from Envigo (Huntingdon, UK) and were supplemented with vitamin mix containing vitamin D_3_ and vitamin K_1_. Detailed diet composition can be found in the Supplement of the related research article [1]. Diets were stored at 4 °C before usage. Food was replaced weekly. Individual body mass was recorded weekly using a portable compact balance (OHAUS Corporation, Model CS200, Parsippany, US). Individual glucose homeostasis was evaluated by glucose tolerance tests every 4-weeks. After 26-weeks of diet, mice were anesthetized using isoflurane inhalation and terminated by intracardiac puncture followed by cervical dislocation. Heart, lung, liver, spleen, interscapular brown adipose tissue (BAT), inguinal white adipose tissue (WAT), and pancreas were isolated, weighted using analytical balance (Denver Instrument GmbH, Model P-114, Goettingen, Germany), and snap-frozen in a 1.5 mL Eppendorf tube using liquid nitrogen and stored at −80 °C. Right and left femora were isolated, length measured with a digital caliper (Starrett, Model EC799A-6/150, Athol, USA), and stored in a 1.5 mL Eppendorf tube at −20 °C in phosphate buffered saline-soaked gauze until further analysis.

### 2.2 Glucose tolerance test (GTT) during long-term high-fat diet

GTT was performed as described before [1]. Food was withdrawn for 5 h for 4-week-old mice, or for 16 h for older mice, prior to GTT. Blood glucose was measured using single-use glucose strips with the ONETOUCH VerioFlex glucometer (LifeScan Europe, Zug, Switzerland; maximum measurement limit 33.3 mmol/L) in samples from tail tip immediately before (time 0) and 15, 30, 60, 90, and 120 min after intraperitoneal injection of sterile 2 g/kg glucose solution (glucose dissolved in tris-buffered saline, pH 7.4). Area under the curve (AUC) was calculated by using the trapezoid rule [6].

### 2.3 *Ex vivo* micro-computed tomography

Micro-computed tomography's of the right femora were performed using Skyscan 1272 (Bruker, MA, US). Prior to micro-computed tomography measurements, femora were thrown at 4 °C overnight. Following day, wrapped femora were taken out of the 1.5 mL Eppendorf tube and placed vertically into a 0.5 mL Eppendorf tube with the proximal end facing upwards and the distal end downwards. Afterwards, 0.5 mL Eppendorf tube was placed ‘up-side-down’ on the respective scanning holder (final femoral scanning position: distal bone end facing upwards and proximal bone end facing downwards). The entire femur was scanned, applying the following scanning parameters a voxel size of 5 μm, a 0.40-degree increment angle, 3 frames averaged, a 66 kV and 142 mA X-ray source with a 0.5 mm Al filter to reduce beam-hardening artefacts. Scans were reconstructed using Skyscan NRecon software (Bruker Optics Inc., Billerica, MA, USA) with correction of X/Y alignment, and identical beam hardening and ring artifact corrections applied to all samples. Afterwards, reconstructed scans were rotated using Skyscan Dataviewer Software (Bruker Optics Inc., Billerica, MA, USA) to ensure 100% vertical positioning of the femora prior to analysis. Trabecular bone was analyzed in reconstructed and rotated scans in a region starting at 0.5 mm proximal of the distal femoral growth plate (to avoid primary spongiosa) and scanning a section spanning 10% of the femoral length in a proximal direction. Trabecular bone was automatically segmented from the inner cortical surface and quantified using the system's analysis software (Skyscan CT Analyser, Version 1.16.1.0). To analyze cortical bone, analysis was performed at the middle of the femur spanning a section of 5% of the femoral length. The software derives outer bone diameter and the diameter of the bone marrow cavity from cross-sectional areas using a circular bone cross-section model. Cortical thickness was calculated as the difference of these two diameters divided by 2. Scanned hydroxyapatite-mimicking phantoms (0.25 and 0.75 g/cm^3^ Ca-HA) were used to allow for bone mineral density (BMD) calculation. Phantoms were scanned by applying the parameters mentioned above and BMD calculations were performed according to Bruker method note “Bone mineral density (BMD) and tissue mineral density (TMD) calibration and measurement by micro-CT using Bruker-MicroCT CT-Analyser”.

### 2.4 Three-point-bending test

Following *ex vivo* micro-computed tomography scanning, right femora were loaded to failure in three-point bending using a Materials Testing System Model 5943 (INSTRON, Norwood, MA, USA). The bones were cleaned off of all muscle tissues and soaked overnight in 1X phosphate-buffered saline (PBS) at room temperature until mechanical testing. Individual femur was placed horizontally on two lower supports with the distal end facing to the left and the proximal end facing to the right side. The distance between the lower supports was 7 mm. The anterior mid-diaphysis was loaded under tension via a third support with a vertical displacement rate of 50 μm/s. Test results were recorded using the system's analysis software Bluehill (Illinois Tool Works Inc., Glenview, IL, USA; Version 3.65). Femoral stress and elastic modulus were calculated as described before [7].

### 2.5 Statistics

Unless stated otherwise, data presented in figures are shown as mean ± standard error of mean (SEM). Statistical differences were assessed using GraphPad Prism version 7.0d (GraphPad Software, San Diego, California, USA). Either two-way ANOVA testing or three-way ANOVA testing with Bonferroni post-test were applied. *P* < 0.05 was considered for all tests significant. For non-linear regression analysis, outliers, detected by ROUT method [3], were excluded from the calculation and are displayed in the figures as stars.

## Ethics Statement

All experiments were approved by the Animal Care Committee of McGill University (Montreal, Canada; protocol number: 2012–7127), complied with ARRIVE guidelines, and conformed to the ethical guidelines of the Canadian Council on Animal Care. Male and female WT and *Col1a1^Jrt/+^* mice were used in this study. Male WT and female WT and OI mice demonstrated diet-induced weight gain during long-term HFD diet, while male OI mice are protected from HFD-induced weight gain [1]. HFD-fed male OI mice did not show any diet effects on organ weights, while female OI mice demonstrated increased WAT weights on HFD. Fragile OI bone phenotype persisted on LFD and HFD. Evaluation of sex-related differences between male and female mice on LFD and HFD demonstrated higher mechanical properties in female WT than male WT mice, while female OI mice showed only higher stiffness than male OI mice.

## CRediT authorship contribution statement

**Josephine T. Tauer:** Methodology, Investigation, Formal analysis, Writing – original draft, Writing – review & editing. **Iris Boraschi-Diaz:** Investigation, Writing – review & editing. **Svetlana V. Komarova:** Conceptualization, Methodology, Funding acquisition, Supervision, Writing – review & editing.

## Declaration of Competing Interest

The authors declare that they have no known competing financial interests or personal relationships which have or could be perceived to have influenced the work reported in this article.
